# A Persistent Interest in Viruses

**DOI:** 10.1371/journal.ppat.1005327

**Published:** 2016-01-14

**Authors:** Samuel Speck

**Affiliations:** Department of Microbiology & Immunology, and the Emory Vaccine Center, Emory University School of Medicine, Atlanta, Georgia, United States of America; University of Florida, UNITED STATES

How is it that individuals choose a career in science? For me, it was exposure to science as a child. My father was an organic chemist and later a carbohydrate biochemist. When I was a young boy, he would bring me to his lab at Michigan State University (MSU), where he would set me up with some small project that mostly involved playing with various pieces of glassware and mixing a few solutions together. After graduating from MSU, I joined the graduate program in Biochemistry at Northwestern University, where I had the good fortune to study with Emanuel Margoliash. My good fortune had less to do with the scientific problem being studied and more to do with the infectious enthusiasm for basic discovery that Margoliash brought to the lab every day. The enthusiastic (but hard-nosed) quest for understanding “how things work” that Margoliash instilled in me has served me well throughout my career.

As my graduate studies were drawing to a close in the late 1970s, the molecular biology revolution was just taking off. DNA and cDNA cloning were now possible, and studies on the development of cancer had revealed that some tumors are caused by viruses, or at least tightly linked to specific viral infections. I joined Jack Strominger’s lab at Harvard University, where I worked with a small group of postdoctoral students studying the human gammaherpesvirus Epstein-Barr virus (EBV) and its ability to transform human B cells in culture. EBV is the etiologic agent of infectious mononucleosis and is also associated with the development of several cancers—most notably, Burkitt’s lymphoma, nasopharyngeal carcinoma, and about a third of Hodgkins lymphomas. Indeed, all characterized gammaherpesviruses (GHVs) are associated with the development of lymphomas—particularly in immunocompromised individuals. An interesting characteristic of herpesviruses is that they establish a chronic infection that cannot be cleared by the host immune system, and in the case of herpesvirus mediated diseases, symptoms are often revealed years after the primary infection. Notably, there is a wide array of herpesviruses that infect humans besides EBV, including those that cause cold sores (herpes simplex virus), chicken pox and shingles (varicella zoster virus), and congenital birth defects (cytomegalovirus).

My research on GHVs, initially focused on EBV, has more recently shifted to a rodent GHV, which has allowed us to use genetics in both the host and the virus to pursue questions about how these viruses maintain a chronic infection in the face of a strong host immune response against it—a key prerequisite for the development of lymphomas and other cancers caused by this family of viruses. My research program is part of a larger effort at the Emory Vaccine Center, where a common theme among many of the labs is the study of pathogens that cause chronic infections. This has led to some important new insights into how chronic infections are maintained. For example, my colleague Rafi Ahmed has shown that during chronic lymphocytic choriomeningitis virus infection in mice, the host immune response becomes ineffective. This finding has been extended to infection of humans with HIV and hepatitis C virus. Notably, key regulatory pathways have been identified, and strategies to awaken the immune response have been developed. While this insight has yet to lead to improved strategies of controlling chronic viral infection, it has allowed a novel means by which to reawaken dormant immune responses to human cancers, such as melanoma and some forms of lung cancer.

What have we learned about chronic herpesvirus infections? The major strategy that these viruses employ to hide from the host immune system is to establish a quiescent infection in a long-lived cell population. More specifically, GHVs infect a specific population of white blood cells, called B lymphocytes—the cells that produce antibodies. The virus drives B cells to both proliferate and differentiate, and ultimately establishes a chronic infection in a specific subpopulation of B lymphocytes (called memory B cells), which are both quiescent and long-lived. Using the rodent model, we determined that during the early phase of infection, the virus actively suppresses host attempts to mount an optimal antibody response—a critical component of the host immune response for controlling most viral infections. This, in turn, facilitates the establishment of a chronic infection. Importantly, we showed that such suppression can significantly impact a coinfection by another pathogen. Thus, coinfection of mice with the rodent GHV and a normally nonlethal rodent malaria strain (*Plasmodium yoelii*) can render the parasite lethal, depending on the timing of the two infections. This finding may be directly relevant to young children in Africa who are simultaneously infected with both EBV and *Plasmodium falciparum* very early in life (i.e., severe malarial anemia may be the result of overlapping acute EBV and *P*. *falciparum* infections).

What are the important problems that remain for GHV biology? We and others are identifying the key regulators encoded by GHVs that are required for establishing a long-lived quiescent infection of specific cell populations. Such regulators represent novel targets for therapeutics that can eliminate or control these infections. Importantly, it is becoming increasingly clear that the development of effective therapeutics and vaccines is predicated on a detailed molecular understanding of the interactions between the host and pathogen. In addition, as a bonus, we get to figure out how it works.

**Image 1:**
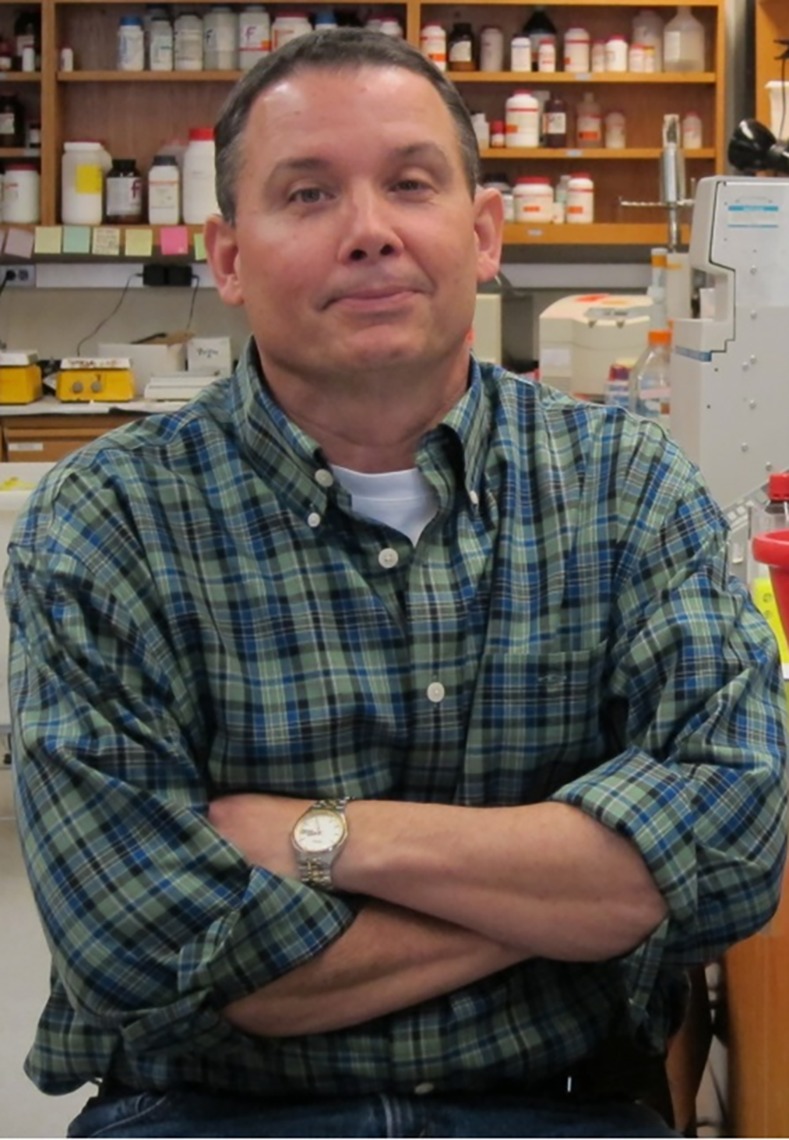
Samuel Speck.

